# Intersectionality of HIV stigma and masculinity in eastern Uganda: implications for involving men in HIV programmes

**DOI:** 10.1186/1471-2458-14-1061

**Published:** 2014-10-11

**Authors:** Gitau Mburu, Mala Ram, Godfrey Siu, David Bitira, Morten Skovdal, Paula Holland

**Affiliations:** International HIV/AIDS Alliance, 91-101 Davigdor Road, Hove, BN3 1RE UK; Division of Health Research, Lancaster University, Lancaster, LA1 4YG UK; MRC/UVRI Uganda Research Unit on AIDS, Entebbe, Uganda; Child Health and Development Centre, Makerere University, Kampala, Uganda; Community Health Alliance Uganda, Kampala, Uganda; Department of Public Health, University of Copenhagen, Copenhagen, Denmark

**Keywords:** Intersectionality, HIV, Stigma, Masculinity, Men, Gender, Disclosure, Peer-support, Uganda, Africa

## Abstract

**Background:**

Stigma is a determinant of social and health inequalities. In addition, some notions of masculinity can disadvantage men in terms of health outcomes. However, few studies have explored the extent to which these two axes of social inequality intersect to influence men’s health outcomes. This paper investigates the intersection of HIV stigma and masculinity, and its perceived impact on men’s participation in and utilisation of HIV services in Uganda.

**Methods:**

Interviews and focus group discussions were conducted in Mbale and Jinja districts of Uganda between June and October 2010. Participants were men and women living with HIV (n = 40), their family members (n = 10) and health providers (n = 15). Inductive analysis was used to identify mechanisms through which stigma and masculinity were linked.

**Results:**

Our findings showed that HIV stigma and masculinity did not exist as isolated variables, but as intersecting phenomena that influenced men’s participation in HIV services. Specifically, HIV stigma threatened masculine notions of respectability, independence and emotional control, while it amplified men’s risk-taking. As a result, the intersection of masculinity and HIV stigma prevented some men from i) seeking health care and accepting a ‘sick role’; ii) fulfilling their economic family responsibilities; iii) safeguarding their reputation and respectability; iv) disclosing their HIV status; and v) participating in peer support groups. Participation in some peer support activities was considered a female trait and it also exacerbated HIV stigma as it implicitly singled out those with HIV. In contrast, inclusion of income-generating activities in peer support groups encouraged men’s involvement as it enabled them to provide for their families, cushioned them from HIV stigma, and in the process, provided them with an opportunity to redeem their reputation and respectability.

**Conclusion:**

To improve men’s involvement in HIV services, the intersection between HIV stigma and masculinity should be considered. In particular, better integration of and linkage between gender transformative interventions that support men to reconstruct their male identities and reject signifiers of masculinity that prevent their access to HIV services, and stigma-reduction interventions that target social and structural drivers of stigma is required within HIV programmes.

## Background

‘Stigma’ is a term that by definition incorporates notions of social exclusion in the context of health and illness. When Earnshaw and Chaudoir [1] propose defining stigma as a social process characterised by exclusion of a person identified with a particular disease, they suggest that stigma is an expression of social values that ultimately determine people’s experiences of illness. In his model of the social construction of diagnosis and illness, Brown [2] illustrates how social beliefs and power relations determine whether an illness is socially acceptable or not.

Parker and Aggleton [3] build on Brown’s model to argue that stigma is not only a product of power relations, but can itself generate or enhance power relations. As a result of these structural dimensions, they argue, stigma is intricately linked to social inequality because it can limit the ability of stigmatised individuals to access important services and institutions patronized by the majority. In other words, stigma can produce inequalities in health through social stratification and exclusion [4].

Consequently, although historically stigma has been conceptualised almost exclusively as a determinant of social exclusion, there have been recent attempts to redefine health inequalities as a derivative of multiple causes of social exclusion, including stigma [5]. This shifting reconceptualisation of stigma as a driver of health inequality, Parker argues, is primarily driven by an increasing understanding of the impact of stigma on people’s access to health services [6].

Applied to HIV, this reconceptualisation helps us differentiate between a social cognitive understanding of stigma – that is, the processes by which people develop negative attitudes towards individuals with HIV – and a structural understanding of stigma – that is, the ways in which power relations inherent in stigma determine who has access to HIV and other health services. This is illustrated by two recent systematic reviews that demonstrate how HIV stigma, through exclusion, isolation and marginalisation, was preventing heterosexual women and men, gay men and sex workers from accessing essential HIV services globally [7, 8].

This reconceptualisation has also been central to an emerging body of work investigating the intersection of stigma with other social determinants of health through the application of intersectionality theory. The intersectionality approach ‘captures lived experiences produced by concomitant, interacting factors of social inequity’ [9], p.272]. It explains how people falling within two or more socially marginalised categories face different and multiple forms of exclusion that intersect to shape their access to essential services [10]. For instance, in a recent review, Monteiro, Villela and Soares [11] demonstrate how the intersection between stigma, social class, gender, race, ethnicity and sexual orientation produces multiple and distinct experiences among people living with HIV in different contexts.

Intersectionality is particularly relevant given the ongoing criticism of public health services that deal with determinants of health separately from each other, despite evidence of associations between them [12]. By failing to account for these interactions, current policies err in reducing these determinants to isolated categories rather than considering them as part of a complex universe of social determinants of health [13]. As a caveat, intersectionality theory does not make *a priori* presumptions about the importance of one category over another [9], nor does it ‘simply add social categories to one another’ to explain inequalities [9], p.276]. Rather, it seeks to demonstrate the convergence of different types of exclusion and marginalisation [9, 10].

Reflecting the need to examine the interaction between HIV stigma and gender, Wyrod [14] presents a conceptual framework for interrogating how the link between HIV stigma and masculinity determines the ways in which men cope with HIV in Uganda. Given that masculinity encompasses what is *believed* to be societies’ expectations of men [15], social norms are prescribed within different cultures that determine how men *ought* to behave, including in the face of illness. Results from studies exploring the relationship between masculinity and men’s health indicate that some masculinity constructs such as success, power and competition protect men from ill health, while others such as risk-taking and self-reliance can predispose men to it [16, 17]. Both stigma and masculinity can affect men’s health-seeking behaviour, so it is critical to examine how these two axes intersect. In this paper, we build on Wyrod’s work [14] by examining this intersection and its perceived influence on men’s involvement in and uptake of HIV services.

## Methods

### Setting and context of the study

This paper presents findings from a qualitative study whose conduct and reporting conforms with the RATS framework [18]. The study was conducted with adult men and women living with HIV in Mbale and Jinja districts of Uganda between June and October 2010. Mbale is a predominantly rural district situated in the eastern region of Uganda, where in 2011 16.6% of men had more than two sexual partners and the HIV prevalence rate among those aged 15–59 was 4.1% [19]. Jinja, on the other hand, is an urban and peri-urban district located on the shores of Lake Victoria in the east-central region of Uganda. In this district, 30.6% of men had more than two sexual partners in 2011, and HIV prevalence among those aged 15–49 was 5.8% [19]. Most residents in this setting live in close-knit communities, with subsistence farming dominating in Mbale, while fishing is common in Jinja [20].

Previous studies of masculinity in these and neighbouring districts claim that masculinity tends to be fixed [21], while unorthodox roles and sexualities, such as gay identities, are frequently contested or rejected [22]. The masculine ideal of being a breadwinner is well established [21], generally advancing the mainstream notion of masculine authority and reputation [23]. In most of Uganda, men dominate better-paying occupations. They are also more likely to attain higher education levels and be employed, compared to women [24].

Jinja and Mbale were among the 40 districts in which the International HIV/AIDS Alliance had implemented a community-based HIV programme known as the Networks project in the four years that preceded the study. The aim of the project was to mobilise and strengthen groups of people living with HIV, and enable them to access HIV services at a time when HIV stigma was rampant [25]. In 2012, we reported how these peer groups of people living with HIV were educating and mobilising their communities to test for HIV and access relevant services [26]. In 2013, we showed how these groups were mobilising their members to challenge and cope with stigma, which was a barrier to their own uptake of HIV services [27]. Data published so far suggest that the project succeeded in mobilising communities of people living with HIV not only to access services but also to participate actively in HIV service delivery; for instance, by challenging stigma, counselling others, providing home-based and palliative care, and referring others to HIV services. However, ‘limited involvement of men’ in service uptake and provision was noted [28], p.352].

### Study aims

The overall aim of the study was to explore the role of community-based peer support groups in HIV prevention and care. In particular, the study sought to establish what motivated people living with HIV in these communities to form or join existing peer support groups; what activities they were involved in; and what challenges, if any, were encountered.

### Study participants

A total of 65 individuals took part in the study, all of them previously involved in the Networks project: 40 were living with HIV, 10 were members of their households and 15 were their health providers. Researchers visited groups of people living with HIV and their family members in their communities, as well as staff working in health facilities providing HIV services to these individuals, and invited them to participate in the study. Researchers provided potential participants with information about the study, including the aims and voluntary nature of their participation. Participants were then given a week to seek clarification and decide if they wanted to participate before providing written consent or a thumb print. The study included key informants purposively selected in order to gain diverse opinions and perspectives on the role of peer support groups. The age range of participating men was 30–64 years, while that of women was 18–52 years. This paper focuses on the themes of stigma and masculinity that emerged from the accounts of both male and female participants. The Science and Ethics Committees of the Uganda Virus Research Institute and the Uganda National Council for Science and Technology granted ethical approval for this study (Table 1).

**Table 1:** **Study participants and methods of data collection**

Methodology	Participant details
In-depth interviews	Key informant interviews with district health officers, district HIV focal persons, district AIDS coordinators, community leaders, medical superintendents of district hospitals and HIV clinic supervisors (n = 15)
	In-depth interviews with people living with HIV who received peer support (n = 10)
Focus group discussions	Focus group discussions with family members from households of people living with HIV (1 session; n = 10)
	Focus group discussions with members of peer support groups of people living with HIV providing peer support to others (3 sessions, n = 30)

### Data collection

Interview guides for in-depth interviews and topic guides for the focus group discussions were developed in reference to existing literature, study aims and a formative pilot phase. Combining interviews and focus group discussions enabled exploration of participants’ perspectives and group dynamics in relation to the study questions, while providing an opportunity for complementary information to be gathered using both methods. Interviews and focus group discussions were performed at locations of participants’ own choosing, usually in a private room at health clinics or in their homes.

Study tools were developed into Luganda, Lusoga and English to accommodate different language preferences of participants. Topics and questions were tailored to each participant group. Interviews lasted 25–50 minutes, while focus group discussions lasted 45–60 minutes. Researchers probed participants’ responses to guide the interviews and allow important issues to be raised by participants themselves. Both interviews and focus group discussions were audio recorded, translated into English as appropriate, and transcribed. Participant recruitment continued until data saturation was achieved. It was through an investigation of the study questions that the topics of stigma and masculinity emerged prominently and gave rise to this paper.

### Data analysis

Interviews and focus group discussion transcripts were subjected to a thematic analysis separately, aided by NVivo 7. Emerging themes were systematically classified and organised in relation to the broad objectives by labelling each line, while remaining open to discovery. By grouping codes into categories and subsequently linking and comparing them to each other through inductive analysis [29], an initial list of thematic codes was generated from interviews and focus group discussions separately, then refined and clustered together based on similarities. Ambiguities were discussed and reconciled by two authors (GM, MR).

## Results

We identified pathways and mechanisms through which stigma and masculinity intersect, and the ways in which they are perceived to affect men’s involvement in and utilisation of HIV services.

### How notions of masculinity affect participation in peer group activities and uptake of HIV services

To understand how masculinity interacts with HIV stigma, we present data related to men’s involvement in support groups. Interviews and focus group discussions overwhelmingly suggested that men "*don’t join the support groups"* and were therefore the groups’ *"biggest challenge*" (in-depth interview #025, woman living with HIV). According to one male focus group participant from Bufumbo Hope group, Mbale, "*Male involvement [was] low, even with married couples",* which seemed to contradict the expectation that married men might be more willing to participate in peer support groups.

Men’s limited involvement in peer support groups is particularly relevant to their uptake of services given that these groups were providing a range of services, including HIV post-test counselling, adherence counseling for antiretroviral therapy, home-based care, and palliative and other psychosocial support. Since men did not join these groups in large numbers, they had little opportunity to provide or receive care. Hence, narratives similar to "*men do not come for HIV tests as much as the women"* (focus group discussion, woman living with HIV, Bufumbo Hope group, Mbale) were common for a range of community-based services:

*Sometimes we organize community education and sensitization sessions, but the men don’t want to come with us.* (In-depth interview #002, woman living with HIV, Abatwogerera PLHIV group, Mutai, Jinja)

When asked why men appeared reluctant to participate in support groups compared to women, participants’ responses often implied that men’s perceptions of manhood were influential. Among the many notions of masculinity apparently influencing men’s health-seeking behaviour and uptake of services, the most prominent were respectability, risk-taking, independence and emotional control. For instance, because peer support groups were involved in activities that were perceived to suit women - such as drama, home-based and palliative care - few men wanted to participate.

When men did join support groups, most preferred to perform physically demanding activities that were deemed more masculine. Hence, in most cases men were involved "*because there are some jobs that we as women cannot do, like building"* (focus group discussion, woman living with HIV, the AIDS Support Organization (TASO) group, Mbale). Therefore, it was common to hear that some groups comprised "*women alone, but later involved men"* and that when *"men were involved, they [carried] out heavy weight tasks*" (in-depth interview #015, woman living with HIV, TASO group, Mbale).

Despite men generally identifying the groups as feminine, opportunities for leadership and training attracted them, since these roles were perceived as compatible with masculine notions of respectability and authority. This was reflected in the narratives that spoke of how the men often chaired groups even when most members were women. Asked how he had got involved in a group’s activities when most men did not, one male participant explained how his wife had *"persuaded [him] to join and [he] became the chairman of that organization"* (in-depth interview #003, man living with HIV, Mbale). In a different interview, in response to questions about the future plans of his peer support group, one participant remarked that *"a skills training institute [was] being started but it is the men who [were] going to train and facilitate it"* (focus group discussion, man living with HIV, Jinja).

Another perception related to men’s participation and uptake of services was linked to the masculine notion of risk-taking. Social expectations for men to be risk-takers emerged from interviews and focus group discussion with people living with HIV and health providers. For instance, narratives were common of how "*men shun using condoms*" (focus group discussion, female household member of people living with HIV, TASO-supported household, Mbale), when consistent condom use is regarded as an important behaviour for preventing HIV acquisition. In addition, it appeared that men were expected to have multiple, often concurrent sexual partners. It was common to hear narratives of how "*men are womanizers; they go around approaching women for relationships"* (focus group discussion, female household member of people living with HIV, TASO-supported household, Mbale). Participants described men who, having discovered they were HIV positive, still continued their promiscuous lifestyle without disclosing their status, and could not join HIV support groups since doing so might have implied their HIV-positive status.

*Most men don’t join [groups] because they still go for other women outside their marriage and they think that when they speak and disclose, they will lose their [extramarital sexual partners]. But without disclosing, they can’t get services.* (In-depth interview # 015, woman living with HIV, TASO group, Mbale)

Another perception related to men’s uptake of services concerned the masculine notion of independence. Participants’ accounts suggested that men often struggled with the idea that they should seek and utilise health services, and accept being linked to long-term care, because as men, they were expected to be physically and mentally strong:

*Women came out for services but men are big-headed and want to be independent, they even refuse to use condoms when they know their positive HIV status; so I don’t know what we can do with the men.* (In-depth interview # 002, woman living with HIV, Abatwogerera PLHIV group, Mutai, Jinja)

Related to this notion of independence were men’s difficulties in adopting a sick role. The need for self-reliance and emotional control commonly emerged as reasons why they rejected this role. For instance, men were neither expected to be, nor were they accustomed to being emotionally dependent on other men for psychosocial support and counselling, although it was generally acceptable to depend on women for care and nurturing within the family:

*You find that some men do not want to come up and join us. They are not used to habits like a man getting counselling from fellow men.* (In-depth interview #022, man living with HIV, Nakaloke, Mbale)

Hence, men struggled to adopt a sick role because this was contrary to notions of masculine independence. Men were described as "*hard to convince"* to take up services, *"even if they were free of charge*" (in-depth interview #017, woman living with HIV, Budondo, Jinja), and were generally "*difficult*":

*It is easy to counsel women; they do what [they are] told to do, but the men are very difficult to counsel and help. They don’t see that they are sick and need our counselling.* (In-depth interview #008, female counsellor, Mutai, Jinja)

### Intersection of HIV stigma and notions of masculinity

On the whole, some signifiers of masculinity, which in our study included physical and emotional strength, respectability and involvement in multiple sexual relationships, seemed to undermine men's health by restricting their participation in peer support groups and, ultimately, utilisation of HIV services. As a consequence, there were *"many people living with HIV who [were] still in hiding, especially men"* (in-depth interview #008, female counsellor, Mutai, Jinja). Our data also suggest that some of these signifiers of masculinity were often intertwined with HIV stigma. For instance, men’s reluctance to adopt a sick role was reinforced by the fact that HIV is a stigmatised disease. It was common for men to wait until they were in advanced stages of HIV disease to seek medical assistance, as one man noted:

*I could be sick and because I do not want people to know that I am sick, I just decide to suffer silently. This is very common in men; most of us suffer silently yet you can only get support if you come out.* (In-depth interview #022, man living with HIV, Nakaloke, Mbale)

While women were not immune to HIV stigma, it appeared that men were particularly known for ‘hiding’ and failing to seek services:

*Men are the ones hiding a lot in the communities. You see men coming in secrecy complaining about certain symptoms.* (Focus group discussion, female household member of people living with HIV, TASO-supported household, Mbale)

Some male participants attributed men’s delay in seeking care to stigma, emphasising that because of stigma, "*silence is very common; our fellow men are out there suffering silently"* (in-depth interview #022, man living with HIV, Nakaloke, Mbale). One mechanism by which HIV stigma was perceived to interact with masculinity to prevent men from accessing services was linked to a sense of shame, secrecy, powerlessness and a loss of respect, qualities that were all contrary to masculine notions of respect. Not surprisingly, it was claimed that when men joined peer support groups they tended to "*hide and disguise themselves as they [participated in] group activities*" (in-depth interview #005, man living with HIV, Jinja). The experience of HIV stigma was perceived to vary between men and women, with men tending to feel more ashamed when diagnosed with HIV:

*Stigma has a way it affects especially men because they don’t normally want to go testing or to join groups; women easily test compared to the men … some men also feel ashamed and powerless because HIV is forcing them to join support groups.* (Focus group discussion, male household member of people living with HIV, TASO-supported household, Mbale)

Another mechanism by which stigma was perceived to interact with masculinity to undermine men’s access to HIV services was related to the notion that men have a social responsibility to provide for their families. This materialistic symbolism of the male as the economic pillar of the household meant that men were perceived to feel particularly ashamed if HIV prevented them from providing for their families through loss of employment and income or physical frailty:

*As men we have an obligation to take care of our families but because of poor health and stigma we are unable to fulfil the family obligations, but irrespective of our status we are expected to provide for our families.* (Focus group discussion, man living with HIV, Positive Men’s Union group, Jinja)

This intersection of socio-economic location and HIV stigma cushioned some participants from the full impact of the latter. When questioned about his experience of stigma, one respondent claimed that he "*was not stigmatised because [he] was doing well financially and supporting [his] family ably* (in-depth interview #013, man living with HIV, Jinja).

To avoid the additional shame of being unable to provide for their families, most men aimed to work and therefore found it difficult to set aside time to attend clinic appointments or participate in group activities, which were generally unpaid. Asked why men were not attending community education sessions delivered by the group, one focus group discussion participant responded: "*men say they have to work"* (in-depth interview #008, female counsellor, Mutai, Jinja). However, some men were quick to defend this situation by illustrating the dilemma they faced when they "*have to take care of [their] families yet also have to contribute towards the fight against HIV by participating in group activities"* (focus group discussion, man living with HIV, Positive Men’s Union group, Jinja). This opinion was also shared by a peer counsellor, who asserted that "*were it not for economic factors, I am sure men would not forget [participating in support groups]"* (in-depth interview #010, male health provider, Mbale).

Our findings suggest that social constructs related to men’s social economic responsibilities not only heightened their sense of shame and stigma, they also prevented men from disclosing their HIV status to their dependents:

*Disclosing to my parents was a problem. I knew they would be very worried because I was the only working man in my family.* (Focus group discussion, man living with HIV, Positive Men’s Union group, Jinja)

Although the link between men’s socio-economic roles as providers for their families and their masculinity seemed to hinge on the notion of men’s social responsibility and respectability, narratives of revived masculinity were encountered, often following treatment, improved physical condition, or ability to get employment and generate income:

*I take the medicine and get energy, I do my work and others see me and are like, "You see the man who was very sick is now performing his family duties". So if others see that, it gives them the courage to disclose without fear of being disrespected or of facing stigma.* (In-depth interview #006, man living with HIV, Mbale)

In other cases, revived masculinity was related to skills-building or livelihood activities that allowed men to generate income as part of a group. Hence, livelihood support was often used to incentivise men to take up services. For example, one participant mentioned how "*the AIDS Support Organization* [TASO, a local non-governmental organisation] *gave Positive Men’s Union a maize mill to encourage men to come out and test so as to access services"* (in-depth interview #011, female health provider, Jinja). Men also agreed that they needed to "*be recognized and given support so that [they] can manage [their] families and on the other hand join with other people to fight stigma"* (in-depth interview #019, man living with HIV, Jinja).

## Discussion

Recent research suggests that some notions of masculinity can disadvantage men in terms of health [16]. Traditional notions of masculinity, such as risk-taking, self-reliance, emotional control, violence and sexual achievement, pose significant risks to men’s health [16, 30, 31] and can also increase their risk of HIV acquisition [32]. The paradox that notions of masculinity can *both* promote and harm men’s health has also emerged from a growing body of literature [23, 33–35]. Our study builds on this literature and on the concept of intersectionality by demonstrating how social constructs of masculinity, such as respectability, risk-taking, independence and emotional control, can intersect with HIV stigma to further disadvantage men’s health, specifically their participation in and utilisation of HIV services.

While previous theory and empirical observations from Uganda have suggested that HIV stigma affects how men cope with HIV [14], what our study adds is a demonstration of how stigma and masculinity may intersect to affect men’s participation in and utilisation of HIV services. Our findings suggest that axes of masculinity and HIV stigma should not be understood as unilateral variables, but as able to amplify or otherwise modify each other to determine how men are involved in HIV services.

Following Parker and Aggleton’s [3] definition of stigma as a social phenomenon that limits the ability of individuals to access important amenities, it appears less contentious to claim that stigmatised groups of people living with HIV may experience inequitable access to health services compared to other members of their communities. This is consistent with the accepted notion that HIV stigma is a form of social marginalisation that causes inequalities in health access [36]. Because men living with HIV in our study setting were subject to stigma, they were already socially disenfranchised compared to HIV-negative men. However, given that levels of stigmatisation can differ even within stigmatised groups, we argue that the extent to which HIV-positive men suffer stigma-related inequity is dependent on prevailing notions of masculinity, such as respectability, risk-taking, independence and emotional control. We therefore assert, based on intersectionality theory, that men’s social identities related to their masculinity may aggravate this inequality. Needless to say, the process by which this could happen is not straightforward. Nevertheless, the crux of intersectionality theory is that by combining the inequality consequences of stigmatisation with the influence of masculinity, it becomes possible to predict that HIV-positive men face *different* inequalities than would be caused by each function in isolation.

This is not to suggest that women are better off. Our paper’s intention is not to compare gender groups but rather to demonstrate that the interaction between two determinants can influence the level of inequality for specific groups. It seems uncontentious to the authors to assume that, overall, women may be more disadvantaged compared to men, at least based on recent research from sub-Saharan Africa [37, 38]. Indeed, other commentators have noted how men, through enactment of their notions of masculine power, can prevent women’s access to and compliance with HIV prevention and treatment [39]. However, our line of argument is that among men, notions of masculinity do have an impact on their real or perceived ability to utilise services. This is particularly relevant given that in our study districts participation of men in HIV care was low [28]. In another study in Mbale district, Byamugisha et al. [35] revealed that poor attendance by men in health clinics was attributed to them "*being busy trying to make ends meet*", and a belief that men who accompany their wives to clinics are ‘*weaklings*’ (p.5).

As notions of masculinity differ across communities, the extent of its impact is likely to differ from place to place. On the one hand, men who live in relatively egalitarian contexts may have less of a need to manage inequalities emanating from their masculinities. On the other hand, interaction of masculinity with other variables, such as culture, social class and sexual orientation, could be significantly determining men’s health. Despite these contextual differences, the potential existence of multiple variables and the interactions between them should be taken into account when designing health interventions. Coburn et al. [12] warn that ignoring one social location over another runs the risk of becoming over-reliant on specific interventions that fail to account for other important drivers of inequality. This is particularly relevant to HIV programmes, whose new interventions should consider socio-culturally constructed barriers to services, such as masculinity, in addition to exclusion from health services based on HIV status.

Mankowski and Maton [40] argue that associations between masculinity and health behaviours could provide *opportunities* to mitigate many social and health problems. They suggest these could occur through gender transformative approaches that challenge those notions of masculinity that endanger men’s health while strengthening others that promote it. Therefore, identifying masculine constructs that promote health-seeking behaviour could be an important strategy for improving men’s health. At the same time, men ought to be empowered to reject harmful constructs that predispose them to ill health through gender transformative interventions. Of course, this differentiation needs to be contextualised and sufficiently nuanced, given that some masculinity constructs can be both protective and harmful [16].

### Implications for HIV programmes

These findings lead us to suggest several practical interventions to mitigate the effects of the intersection of stigma and masculinity. First, HIV programmes should stimulate community conversations [41] to educate men and women about the possible harmful effects of adhering to prevalent masculine notions of risk-taking, independence and emotional control. Community discussions should aim to change gender attitudes, challenge stereotypical gender roles and their related gender inequities, and increase help-seeking and uptake of protective sexual behaviours [42].

Second, HIV programmes and peer support groups should better integrate social protection and livelihood interventions targeting HIV-positive men and their families. Repositioning peer support groups as means to helping men achieve responsible fatherhood and respectability could increase their involvement. Men in our study were willing to leverage notions related to responsible and respectable fatherhood to participate in livelihood activities linked to peer groups, and in the process circumvent HIV-related impoverishment and subsequent stigma. In South Africa, men were willing to disclose their status and take up HIV treatment when it was made clear that doing so would enable them to return to work and provide for their families, and in the process, command respect from their communities [43, 44].

Third, interventions that strengthen HIV-positive men’s social support networks, such as their families, close friends and peers, should be bolstered to help men cope with stigma [45]. This is particularly important given the inverse relationship between social support and perceived stigma in Uganda [46]. In addition, engaging men and women living with HIV to openly challenge stigma [27], could reduce instances of enacted and perceived stigma. Within this, the use of a role-modelling approach, leveraging the few men who are already actively participating in peer-support groups, could be implemented to support other men cope with stigma, reject harmful masculine constructs, and participate in peer support groups and HIV services.

## Conclusions

Understanding the intersection of HIV stigma and masculinity provides important insights into inequalities that may exist in regard to men’s participation in and utilisation of HIV services in Uganda, and could inform HIV programmes as they seek to better engage men in HIV care. Specifically, our findings suggest that there is a need to link interventions that transform notions of masculinity by supporting men to reconstruct their male identities and reject harmful normative notions of masculinity [47] with those that target social and structural drivers and facilitators of HIV stigma at the individual, family, community and institutional levels.

However, before making firm conclusions regarding the nature of the interaction between HIV stigma and masculinity, the limitations of our findings should be noted. Because our study was exploratory, we do not know if or how the relationship between HIV stigma and masculinity differs across socio-demographic groups, geographic locations or time. In addition, because we did not collect detailed information regarding participants’ education, marital status, religion, culture, sexual orientation, employment and so on, the contextualisation, interpretation and transferability of our findings is somewhat limited. Nevertheless, our study operationalises earlier suggestions concerning the need to understand intersectionality’s influence on inequality [10].

In addition, we emphasise that the constructionist paradigm that informs our findings of how masculinity is represented and interpreted does not claim causation. Hence, straightforward inferences related to how masculinity and stigma influence *individual* men’s behaviour cannot be made from our data. The most we claim is that the intersecting stigma and masculinity narratives we identify in our study constitute powerful symbolic resources that are likely to influence the meaning that men *collectively* ascribe to peer support groups, their activities and services.

The primary purpose of our study was not to examine masculinity but rather peer-support groups, yet the concept of masculinity in the context of HIV stigma and peer support groups emerged as a strong theme in respondents’ accounts. A deeper understanding of how masculinity is constituted, performed and experienced in the sample would have aided a more nuanced interpretation of the data, and may have allowed us to examine in detail the implications for men’s reputations of stepping out of orthodox masculine roles, including in relation to their perceived sexual identities. Despite these limitations, our findings still provide useful information related to masculinity in the context of stigma: a convergence that is rarely explored in the literature.

While intersectionality theory is a useful approach to better understanding the confluence of social locations that shape inequalities, it is not flawless. For example, while multiple interactions could exist, the theory does not prescribe which axes to consider, but leaves researcher to decide [48]. Nevertheless, there is a growing consensus that intersectionality, with its ability to incorporate multiple social determinants, is an approach that can successfully inform HIV and other public health interventions.
